# Validity and reliability of the ‘Isometric Exercise Scale’ (IES) for measuring ratings of perceived exertion during continuous isometric exercise

**DOI:** 10.1038/s41598-021-84803-8

**Published:** 2021-03-05

**Authors:** John W. D. Lea, Jamie M. O’Driscoll, Damian A. Coleman, Jonathan D. Wiles

**Affiliations:** School of Human and Life Sciences, Canterbury Christ Church University, Kent, UK

**Keywords:** Physiology, Circulation, Hypertension

## Abstract

Isometric exercise (IE) interventions are an effective non-medical method of reducing arterial blood pressure (BP). Current methods of prescribing and controlling isometric exercise intensity often require the use of expensive equipment and specialist knowledge. However, ratings of perceived exertion (RPE) may provide a more accessible means of monitoring exercise intensity. Therefore, the aim of this study was to assess the validity of a specific Isometric Exercise Scale (IES) during a continuous incremental IE test. Twenty-nine male participants completed four incremental isometric wall squat tests. Each test consisted of five 2-min stages of progressively increasing workload. Workload was determined by knee joint angle from 135° to 95°. The tests were continuous with no rest periods between the stages. Throughout the exercise protocol, RPE (IES and Borg’s CR-10), heart rate and blood pressure were recorded. A strong positive linear relationship was found between the IES and the CR-10 (r = 0.967). Likewise, strong positive relationships between the IES and wall squat duration (r = 0.849), HR (r = 0.819) and BP (r = 0.841) were seen. Intra-class correlation coefficients and coefficients of variations for the IES ranged from r = 0.81 to 0.91 and 4.5–54%, respectively, with greater reliability seen at the higher workloads. The IES provides valid and reliable measurements of RPE, exercise intensity, and the changes in physiological measures of exertion during continuous incremental IE; as such, the IES can be used as an accessible measure of exercise intensity during IE interventions.

## Introduction

Hypertension, characterised by a sustained elevation in arterial blood pressure (≥ 140 mmHg systolic and/or ≥ 90 mmHg diastolic), is the leading attributable risk factor for increased CVD mortality^1^. Exercise has been recommended as a non-pharmacological lifestyle modification for the treatment of hypertension^2^. Isometric exercise training (IET) interventions have been shown to be an effective and time efficient methodology to reduce resting^3–6^ and ambulatory blood pressure^6^.

The control of exercise intensity is a key factor in ensuring the safety and efficacy of physical activity in any context, including athletic, recreational, and therapeutic settings^7,8^. Previous methods of administering IET and monitoring its intensity have tended to require expensive equipment such as isokinetic dynamometers^3,9,10^, hand grip dynamometers^11^ and electromyography (EMG)^5^. It has been suggested that the need for expensive equipment and time-consuming testing protocols, may present unnecessary barriers that could ultimately limit the effectiveness of these interventions^12^. Consequently, more accessible modes of IET that could be implemented in the home have been explored. One such intervention, is the use of the isometric wall squat, where intensity is controlled by manipulation of the knee joint angle^13^. A 4-week home based isometric wall squat intervention, with exercise intensity derived from a maximal isometric wall squat test^6,14^, was shown to produce significant reductions in resting^6,15^ and ambulatory arterial BP^6^. While these methods of training are more accessible than previous iterations, the ability to accurately monitor exercise intensity without laboratory testing and the use of additional equipment could help to further promote uptake of this type of IE intervention.

Ratings of perceived exertion (RPE) could provide an easy to use and accessible alternative means of assessing and monitoring exercise intensity^16,17^. Indeed, it has long been established that RPE provides an accurate estimation of exercise intensity and physiological exertion during cardiovascular exercise^18^. In addition, there is now a growing body of evidence that indicates that various RPE scales provide a valid measure of exercise intensity during resistance exercise, including the Borg 6-20^19^, Omni-res^20^, and the Borg CR-10^21^ scales. The validity of these scales has been shown to be independent of participant age^22,23^ or sex^21,24,25^. Additionally, the Borg CR-10 scale has largely been adopted within IE research up to this this point^14^, despite its intended application being for rating pain with no numerical ceiling effect^26^. In an exercise setting, where the average participant’s understanding of RPE is likely to be limited, the open-ended nature of the CR-10 scale may make monitoring and prescribing IE intensity more difficult. There are currently no RPE scales that have been specifically designed and validated for IET; it has been suggested that it is important to design and validate scales for specific populations, exercise types and modalities^16^, and that caution should be taken when using RPE scales with modalities and materials other than those they have been validated for^27^. It has also been proposed that for an RPE scale to be considered a valid measure for use in the clinical and/or health-fitness setting, it must demonstrate both concurrent and construct validity, evidenced by strong positive correlations with physiological variables (e.g. HR) and a previously validated criterion scale respectively^28^.

Therefore, the aims of this research were to: (1) assess the construct validity of a new isometric exercise scale (IES) as a measure of RPE during isometric wall squat exercise, using the frequently adopted CR-10 scale as a criterion measure; (2) examine the validity of the IES to measure changes in isometric wall squat intensity during a continuous maximal incremental test; (3) explore the concurrent validity of the IES using criterion measures of physiological exertion (HR and BP); and (4) examine the reliability of the IES responses over time.

## Methods

### Participants

Twenty-nine normotensive male volunteers (age: 23.2 ± 4.0 years; stature: 180.9 ± 7.8 cm; body mass: 82.7 ± 17.3 kg) participated in this research. All participants were physically active, non-smokers and not taking any medication that could affect the study results. Furthermore, participants self-reported that they were not suffering from any injury or disease. Written informed consent was given by all participants and they agreed to maintain their regular exercise and dietary habits between testing sessions and for the length of study.

### Study design

All participants were required to attend the laboratory on four separate occasions, separated by a minimum of 48 h. Each session followed the same procedures; starting with resting measurements before completion of a continuous maximal incremental wall squat tests. Participants were asked to abstain from food 4 h, caffeine 12 h, alcohol and strenuous exercise 24 h pre-testing. All participants verbally confirmed adherence to the testing requirements prior to the start of each testing session. This study was approved by Canterbury Christ Church University’s Ethics Committee (15/SAS/223) and conducted according to the 1964 Declaration of Helsinki.

### Procedures

#### Familiarisation

Prior to the first testing session participants received an information pack outlining the testing protocols and measurement procedures included in the study. At the start of the first laboratory session participants had the study design, resting and exercise measurements, and exercise protocols explained to them verbally. As part of this explanation, participants were shown the equipment that would be used and were given a demonstration of the wall squat, including the correct wall squat position. Finally, participants were shown the RPE scales and received the standardised instructions and anchoring. Following this, if the participant wished to be part of the study, written informed consent was collected and resting measurements were taken.

#### Resting measures

Upon arrival to the laboratory, participants rested in a seated position for 10 min. After 10 min rest, HR, systolic BP (SBP), diastolic BP (DBP) and mean arterial pressure (MAP) were recorded using an oscillometric BP monitor on the participants left arm (Dinamap^®^ Pro, GEMedical Systems, Slough, UK). Three measurements were taken, each separated by 1-min^29^. Following the seated measurements, participants rested in a supine position for 15 min. After an initial 10-min period, HR and BP were measured continuously for 5 min using a plethysmographic device (Task Force^®^ Monitor, CNSystems, Graz, Austria). Resting HR and BP values were calculated as the mean of the 5-min supine measurement period.

#### Maximal isometric wall squat test protocol

**Figure 1:**
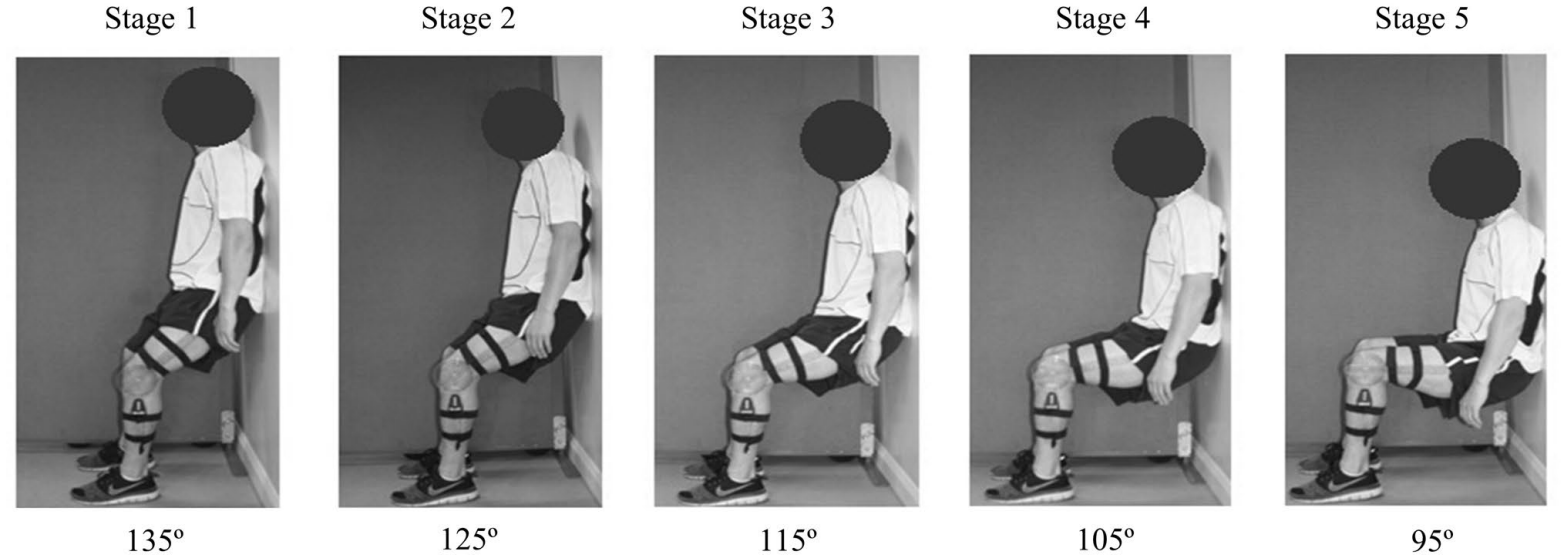
Knee joint angles used for the five consecutive 2-min stages of the incremental isometric exercise test (left to right: 135°, 125°, 115°, 105°, and 95°).

Following the resting measures, participants were required to complete a maximal incremental isometric wall squat test, as described in^13^. The test required participants to lower their back down a fixed wall and make small adjustments to their feet position until the required knee joint angle was reached, while maintaining a vertical lower limb and an erect trunk. Knee joint angle was measured using a clinical goniometer, secured to the participants lower and upper leg using elasticated Velcro strapping. The fulcrum was aligned with the lateral epicondyle of the femur, the moving arm was placed on the lateral midline of the femur using the greater trochanter for reference and the stationary arm on the lateral midline of the fibula using the lateral malleolus and fibular head for reference. A spirit level was attached to the stationary arm to ensure that the lower leg was kept vertical during exercise. The internal angle between the femur and fibula was measured^13^. The test consisted of five consecutive 2-min stages, beginning at a knee joint angle of 135° and guided to reduce the angle by 10° every 2 min (125°, 115°, 105°, and 95°) (Fig. 1). Each participant’s foot position was measured from the back of the left heel to the wall and their back position was measured as the distance from the ground to the lower back, which was defined as the lowest point of contact that the participants back had with the wall. Participants were not permitted to stand or rest between angles. The test continued until volitional exhaustion, the participant was unable to maintain the required knee angle, or completion of the 10-min test^8^. Participants were monitored were monitored for signs of physical distress throughout the test and were instructed to terminate the exercise at the first signs of dizziness or feeling unwell. Verbal encouragement was given throughout, with instructions to maintain normal breathing to avoid the Valsalva manoeuvre. Heart rate and BP were recorded continuously during the test; mean HR and BP (SBP, DBP and MAP) were calculated for the last 5 s of each minute of the test.

#### Ratings of perceived exertion

**Figure 2:**
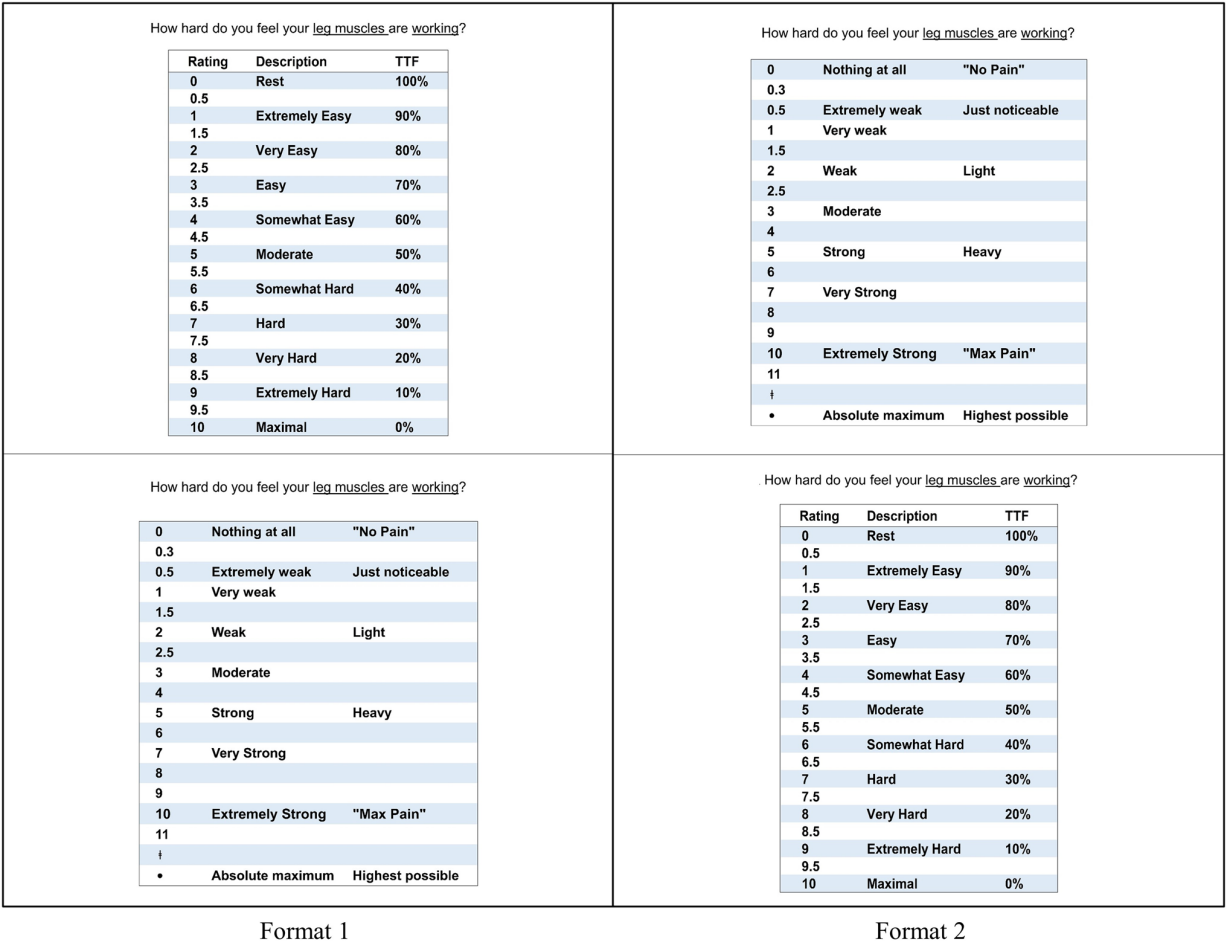
RPE scales presentation formats.

Participants were asked to rate the perceived exertion in their active muscles using the IES and Borg’s CR-10 scales, 50 s into each minute of the test. Participants were cued to give their ratings using the standardised questions: “How hard do you feel your leg muscles are working”. The participants were randomly assigned into one of two groups: Group 1 were asked to rate their perceived exertion using the IES first followed by the CR-10; group 2 rated using the CR-10 first and then the IES. The scales were in full view of the participant for the entire test, presented one above the other, either IES on top or CR-10 on top (Fig. 2) depending on group allocation. The scales were presented in this way, rather than side-to-side, to stop participants picking a rating on the first scale and then moving sideways to the corresponding value on the second scale, without consideration of differences in the two values.

#### Scale instructions and anchoring

Standardised scaling instructions and anchoring procedures were explained for both scales before testing. The standardised instructions and anchoring procedures for the CR-10 scale were used, as previously published in Borg’s work^26^. The instructions for the IES were as follows: “This scale is used to rate how hard you think your active muscles are working. This scale has 3 different columns: Rating, Description and TTF. The ‘Rating’ numbers are from 0–10 and are used to rate the exertion or effort in the active muscle group(s). The ‘Description’ words and 'TTF' are used to help you choose a rating. 0 (Rest) is absolutely no effort, as felt during complete rest. 5 (Moderate) is right in the middle of 0 and 10. It’s not especially hard and it is no problem to continue; but it no longer feels comfortable. 10 (Maximal) is maximum effort; your muscles are working as hard as they can, and you can only maintain this for seconds before you will have to stop.

TTF (Time to Failure) indicates the amount of time remaining, during an isometric contraction, before you will be unable to continue. In other words, this describes how much you have left in your 'fuel tank'. 100%—your muscles are fresh; you haven’t started the contraction yet (fuel tank is full). 50%—means you can continue to hold the contraction for the same amount of time that you have already completed (fuel tank is half full). 0%—your muscles are failing/have failed (fuel tank is empty). When you give your rating; focus only on the muscle group(s) that is working. You can use the ‘Description’ words, the Time to Failure (TTF), and/or you can simply rate the exertion out of ten”.

### Data analysis

All data were analysed using the statistical package for social sciences (SPSS 22 release version for Windows; SPSS Inc., Chicago IL). Before analysis, all data were checked for conformity with the parametric assumptions^30^. Construct validity between the IES and CR-10 ratings was determined using linear regression analysis and Spearman's Rank-Order Correlation. Concurrent validity of the IES and CR-10 results with the criterion measures of exercise intensity was assessed using Spearman's Rank-Order Correlations. To test for differences in concurrent validity, between the IES and CR-10, validity coefficients underwent Fishers Z score transformation followed by statistical analysis for differences in the Z-Scores. Reliability of the IES, CR-10, HR and BP results across the four testing sessions were examined separately using: two-factor (Session × Intensity) repeated measures ANOVA’s or Friedman’s test (normal distribution dependant); Intraclass Correlation Coefficients (ICC); Standard Error of Measurement (SEM) and Coefficient of Variations (COV). For the difference tests, the ‘Session’ factor had four levels (testing sessions 1–4), and the ‘Intensity’ factor had five levels (knee angle—135°, 125°, 115°, 105° and 95°). Where main effects were found, post-hoc testing was conducted with Bonferroni adjustment for multiple comparisons. The ICC (3,1)^31,32^ model was used to assess the agreement between the repeated measures taken during consecutive sessions. Within-participant variance was calculated as the SEM from the ICC analyses, and as COVs with 95% confidence interval, derived from log-transformed two-way ANOVA for each variable^30,33^. ICC and COV results, for the IES and CR-10 scales, were considered to be significantly different if the mean results for each scale lay outside of the 95% confidence interval of the other. An alpha level of < 0.05 was set as the threshold for statistical significance. All data are expressed as mean ± S.D. unless otherwise indicated.

## Results

### Resting measures

At the start of each of the four testing sessions, resting measures were recorded for each participant. The mean resting values for HR, SBP, DBP and MAP were: 61 ± 8 b min^−1^, 109 ± 7 mmHg, 63 ± 5 mmHg, and 80 ± 6 mmHg, respectively. There were no significant differences in any resting measures between trials (P > 0.05). The Intra-class correlation coefficients for the resting measures ranged from r = 0.52 to 0.91. Coefficients of Variation (with 95% confidence intervals) were 4.0% (3.5–4.9%) for resting HR, 2.9% (2.5–3.5%) for SBP, 6.2% (5.3–7.6%) for DBP, and 4.2% (3.6–5.1%) for MAP.

### Construct validity

**Figure 3:**
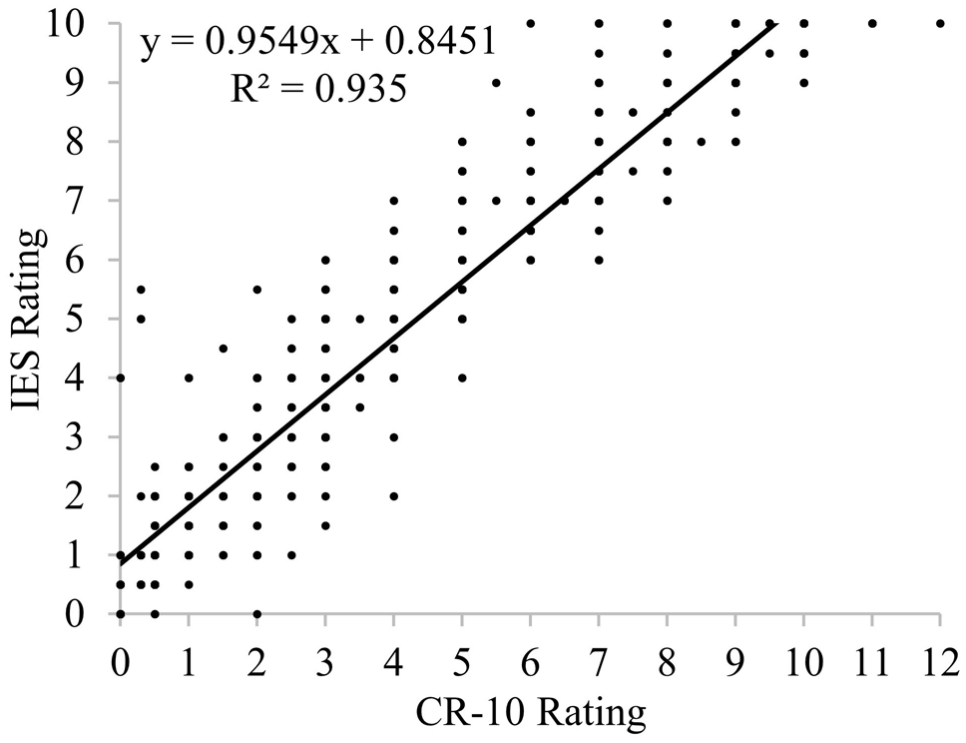
The relationship between the IES and CR-10 ratings of perceived exertion.

The construct validity of the IES was established by correlation and linear regression analysis of the ratings from the IES and CR-10 scale. Spearman's Rank-Order Correlation showed a strong positive linear relationship (r = 0.97, P < 0.001) between the CR-10 and IES ratings of exertion. Likewise, linear regression analysis to assess the ability of the CR-10 results to predict the IES results, showed a significant linear regression equation (F (1, 977) = 13,958, P < 0.001) with an r value of 0.97 (Fig. 3).

### RPE and exercise intensity

**Figure 4:**
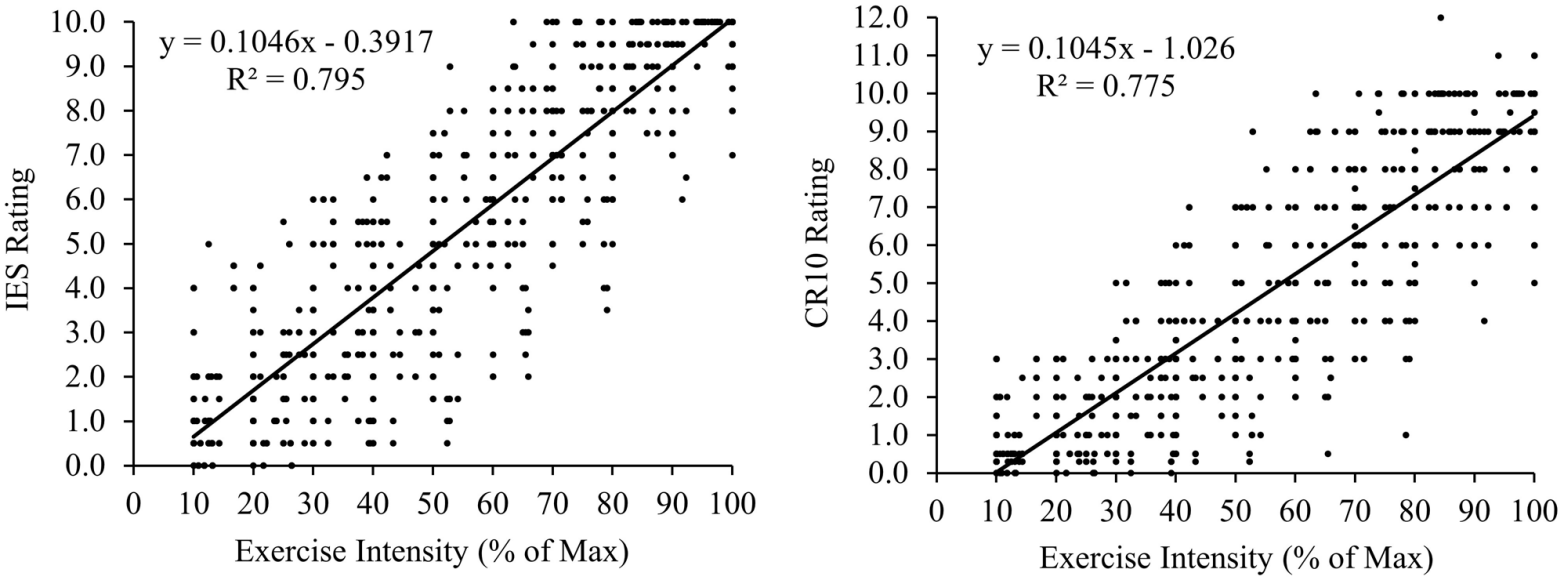
The relationships of the IES and CR-10 ratings with isometric exercise intensity (as a percentage of maximum).

The validity of the IES and CR-10 to represent isometric exercise intensity was assessed by correlating the RPE ratings against percentage of maximum exercise intensity (workload × wall squat duration). Strong positive correlations were shown for the IES (r = 0.89, P < 0.001) and CR-10 (r = 0.88, P < 0.001) with exercise intensity (Fig. 4).

### RPE and measures of physiological exertion

**Figure 5:**
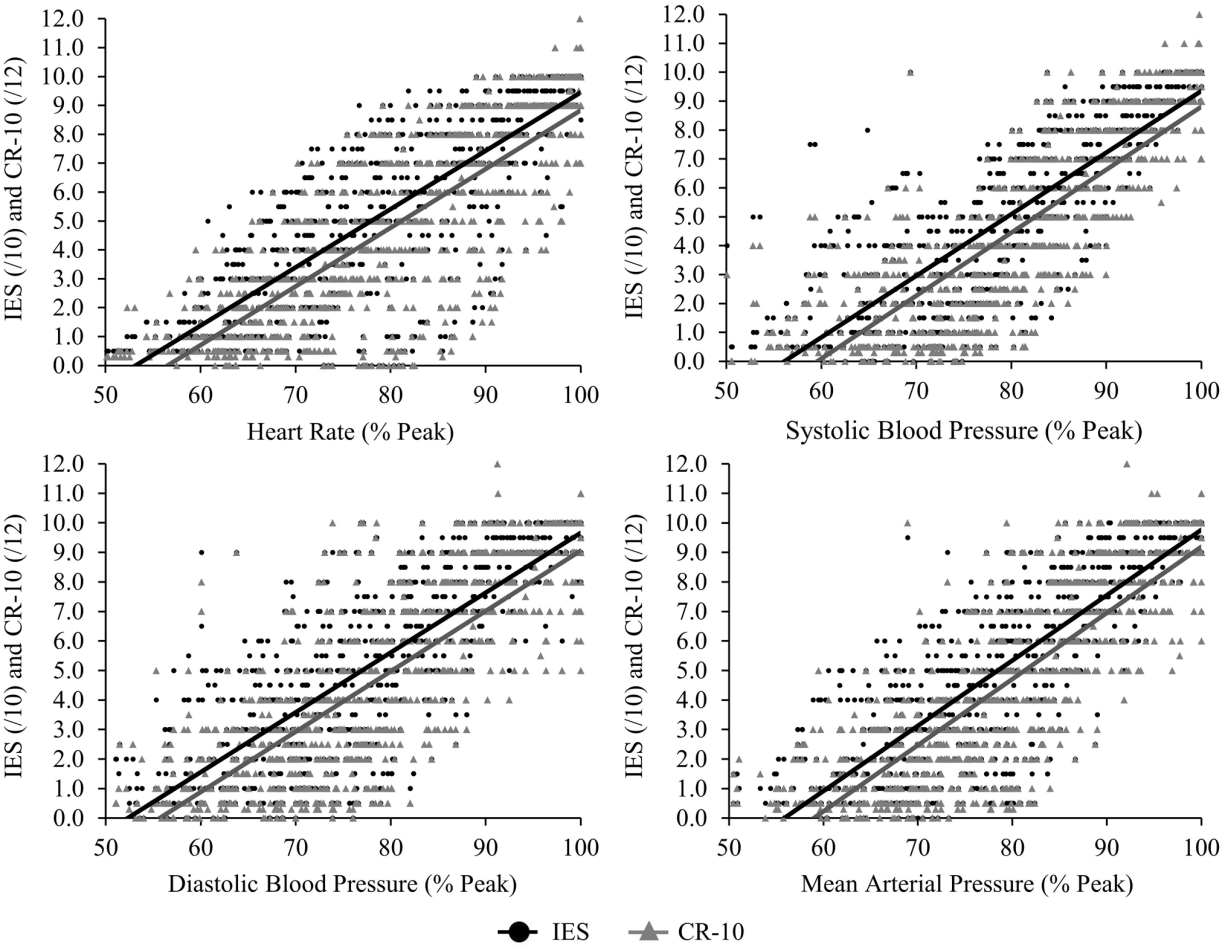
The relationships of the IES and CR-10 ratings with the physiological measures of exercise intensity.

Significant positive relationships were found when correlating the physiological measures of exercise intensity: HR (r = 0.82 and r = 0.81, P < 0.001), SBP (r = 0.84 and r = 0.84, P < 0.001), DBP (r = 0.81 and r = 0.80, P < 0.001), and MAP (r = 0.84 and r = 0.83, P < 0.001) with RPE ratings from the IES and CR-10 respectively (Fig. 5).

### Reliability of exercise measures

There were no significant differences in IES or CR-10 ratings between sessions at any knee angle (P > 0.05). The ICCs and COVs between sessions 1 and 2, fell outside of the confidence intervals for the between sessions 2–4 comparisons; therefore, the reliability data for sessions 1 and 2 are presented separately to sessions 2–4. ICCs for sessions 1 and 2, across all knee joint angles, ranged from r = − 0.49 to 0.76 (SEM = 0.67–1.07) and r = 0.30–0.76 (SEM = 0.71–1.39) for the IES and CR-10 respectively. Additionally, the COVs between sessions 1 and 2 ranged from 42.1 to 10.5% for the IES and 77.2–12.1% for the CR-10. The ICCs for sessions 2–4 ranged from r = 0.81 to 0.91 and r = 0.79–0.90 for the IES and CR-10 respectively (Table 1). The ICC for the 135° knee angle was significantly higher for the CR-10 scale when compared to the IES; no other differences in ICCs were found. The SEMs calculated for Sessions 2–4 ranged from 0.37 to 0.87 for the IES and 0.35–0.84 for the CR-10 Scale. As such, The COVs ranged from 54 to 4.5% and 41.9–7% for the IES and CR-10 respectively, with the lowest variances seen at the higher intensity levels (Table 1).

**Table 1:** IES and CR-10 results at each knee angle between sessions and the corresponding reliability statistics.

	Knee angle	Session Number				ICC	SEM	COV
		1	2	3	4			
IES	135°	1.9 ± 1.5	1.4 ± 1.3	1.3 ± 1.1	1.7 ± 1.5	0.81 (0.70–0.89)	0.67	54.0% (44.9–70.0%)
	125°	3.9 ± 1.8	3.5 ± 1.8	3.3 ± 1.9	3.7 ± 2.2	0.86 (0.78–0.92)	0.87	33.1% (27.8–42.1%)
	115°	6.6 ± 1.7	6.2 ± 2.0	6.1 ± 2.0	6.1 ± 2.3	0.91 (0.85–0.95)	0.72	14.2% (12.0–17.6%)^b^
	105°	8.6 ± 1.2	8.5 ± 1.2	8.2 ± 1.4	8.1 ± 1.5	0.83 (0.72–0.91)	0.56	8.1% (6.8–10.4%)^b^
	95°	9.4 ± 0.5	9.3 ± 0.8	9.3 ± 0.8	9.1 ± 1.0	0.84 (0.66–0.93)	0.37	4.5% (3.5–6.7%)^b^
CR-10	135°	1.2 ± 1.0	1.0 ± 1.0	0.9 ± 0.9	1.0 ± 0.9	0.90 (0.83–0.94)^a^	0.35	41.9% (35.0–53.7%)^b^
	125°	3.0 ± 1.7	3.0 ± 1.7	2.7 ± 1.8	3.0 ± 2.1	0.87 (0.78–0.92)	0.76	38.9% (32.6–49.7%)
	115°	5.7 ± 2.3	5.4 ± 2.1	5.3 ± 2.3	5.4 ± 2.5	0.90 (0.83–0.94)	0.84	18.7% (15.9–23.5%)
	105°	7.6 ± 1.8	8.0 ± 1.5	7.5 ± 1.8	7.5 ± 1.7	0.79 (0.64–0.88)	0.79	13.7% (11.4–17.7%)
	95°	8.6 ± 1.1	9.0 ± 1.2	9.0 ± 1.5	8.9 ± 1.2	0.86 (0.69–0.94)	0.48	7.0% (5.4–10.3%)^b^

*ICC* intraclass correlation coefficients (95% confidence intervals), *COV* coefficient of variation (95% confidence intervals). ICC and COV values for comparisons of sessions 2, 3 and 4 only.

^a^Significantly greater ICC value than the other RPE scale at the same knee angle.

^b^Significantly lower variance than the other RPE scale at the same knee angle.

## Discussion

This study demonstrated that the IES is a valid and reproducible measure of RPE, exercise intensity and the physiological responses to isometric wall squat exercise. The construct validity of the IES was assessed using the Borg CR-10 scale, which has previously been shown to be a valid and reliable measure of RPE and exercise intensity during resistance exercise^34^ and is the scale most commonly adopted for this type of exercise intervention^14^. The IES ratings showed excellent agreement with the ratings from the Borg CR-10 (r = 0.97), during correlation and linear regression analysis. In addition, there were no significant differences in the relationships shown with exercise intensity and physiological measures of exertion (HR and BP) between the IES and CR-10 scales. The Borg CR-10 scale has previously been used to validate the OMNI Elliptical Ergometer Scale, during aerobic exercise, yielding similar construct validity coefficients to the present study (r = 0.96–0.98)^28^. During dynamic resistance exercise, the CR-10 scale was also used to validate a novel Estimated Repetitions to Failure Scale, giving strong validity coefficients ranging from r = 0.86 to r = 0.96, depending on the specific exercise used^35^. Likewise, the now widely used OMNI-RES scale was validated during resistance exercise using the Borg 6-20 scale^36^; this analysis showed validity coefficients from r = 0.94–0.97. The construct validity results of the current study, when compared to previous research, suggest that the IES is a valid measure of RPE during isometric exercise.

Ratings from the IES were shown to be an accurate representation of exercise intensity during maximal isometric wall squat exercise. This was shown through correlation of the IES results with wall squat duration, which in this case represents an increase in both contraction time and workload throughout the test. The results of this analysis showed a strong positive relationship between the two variables (r = 0.89). Previous research has shown the OMNI-RES scale to be a valid measure of exercise intensity, with correlations ranging from r = 0.89 to 0.91 in males and females^27^. Similarly, the CR-10 was shown to be a valid measure of exercise intensity with validity coefficients of r = 0.77 at baseline and r = 0.91 following a 12-week training intervention^37^. Likewise, in production mode, the Borg 6-20 scale has also been shown to be valid when used by sedentary, active and strength trained individuals alike (r = 0.83–0.92)^38^. As such, the validity coefficient shown for the IES is comparable to those shown in previous resistance exercise research, suggesting it is an accurate measure of exercise intensity.

The IES also showed strong positive relationships with HR (r = 0.82) and BP (r = 0.81–0.84), indicating that the IES can accurately represent the changes in physiological exertion caused during the incremental isometric test, to the same extent as the CR-10 scale. The CR-10 scale has previously been shown to produce strong positive correlations with HR (r = 0.76) and blood lactate (r = 0.730) during dynamic weight training^39^, and with HR (r = 0.71) during bodyweight suspension training^40^. Likewise, the CR-10 has shown comparable relationships during aerobic training^18^. The strong relationships seen between the IES and physiological measures could allow it to be used as an important additional safety measure during IET, to ensure that participants are working at intensities that keep them within safe HR and BP limits^8,41^. However, this requires further investigation for confirmation.

Reliability of the IES measures across the four testing sessions was examined by correlating the results from consecutive sessions using intraclass correlation coefficients. The ICC measures of reliability between sessions 1–2 were significantly different, when compared to between sessions 2–3 and 3–4. These results indicate a learning effect following the first session and suggests that habituation with the isometric wall squat exercise and RPE scale may be required before a stable relationship is achieved. The ICC results for the IES showed excellent agreement (r = 0.81–0.91) across sessions 2–4, indicating that the IES is a reliable measure of RPE and exercise intensity over time. These reliability coefficients were closely matched by the ICCs for HR and BP, suggesting that the relationship between the IES results and physiological exertion is stable over time. These results are comparable to those shown previously for the OMNI-RES Thera-band (r = 0.72–0.76)^42^ and Borg CR-10 (r = 0.88)^43^ scales during different forms of resistance exercise. Additionally, when the OMNI-RES scale was used in production mode, where the participant selects/modifies the exercise intensity to elicit a specific RPE response, similar reliability coefficients were found (r = 0.69–0.95)^44^.

The within participant variance was assessed using the SEM and COV. The CR-10 scale showed lower variance at the 135-workload (0.35 vs 0.67) when compared to the IES, corresponding to the significantly higher ICC result seen with the CR-10 at that level. Whereas, the IES showed lower SEMs at the four higher workloads, when compared to the CR-10. This translated to statistically lower COVs at the three highest workloads with the IES. The COV results for the IES showed higher percentage variance at the lighter intensities and lower variances at the higher intensities; this is to be expected as the lower average RPE values seen in the earlier stages of the test (IES = 1.6 and CR-10 = 1.0 in the first stage) mean that even the smallest possible change between session (0.5) would elicit 30–50% variance. Arguably, this high variance makes comparison of COVs from RPE with COVs from other measurement methods, e.g. HR, inappropriate; however, this can still be a useful measure of the variance/reliability when comparing two like measures, such as two RPE scales. The CR-10 scale produced significantly lower variance at the lowest intensity (135°), possibly due to the increased number of lower value numbers and therefore smaller differences between values at the lower end of that scale (0, 0.3, 0.5 and 1), whereas the IES showed significantly lower variance at the highest three intensities (115°, 105° and 95°), possibly due to the simpler closed-ended nature of the IES as opposed to the open-ended CR-10. Since these intensities are more representative of the intensities used during IET for reducing resting blood pressure^14^, this may suggest the IES is more appropriate during this type of intervention. Furthermore, previous analysis of the reliability of the CR-10 showed a COV of 17%, for exercise eliciting an average RPE of approximately six for females and seven males^45^. This RPE is approximately equivalent to the mean IES results seen at the 115° knee angle (6.3) which showed between session variance of 14.2%. Similarly, Day et al.^43^ assessed the reliability of the CR-10 across 2-sessions at three difference intensities, giving a COV of 14.5%. The three intensities used in Day’s study gave mean RPE scores of 3.7, 5.6 and 6.9, approximating the mean IES ratings achieved across the 125° and 115° knee angles in the current study (3.6–6.3); The IES COVs for these knee angles ranged from 14.2 to 33.1%. These results suggest that the IES is reliable across sessions following habituation, especially at the higher workloads associated with BP training interventions.

Further research is required to explore the validity of IES during discontinuous isometric exercise at submaximal intensities, as is currently used during isometric wall squat training for the reduction of resting blood pressure^15^. Additionally, research is needed to explore the potential use of RPE as a means of prescribing and monitoring IET intensity, especially in pre-hypertensive and hypertensive populations.

## Conclusion

The IES provides a valid and reliable measure of RPE and exercise intensity during maximal isometric wall squat exercise. Additionally, the IES results produced strong positive relationships with the criterion measures of physiological exertion (HR and BP). As such, the IES can be used as a valid measure of RPE and could be useful in the selection and monitoring of workloads during IET interventions for the reduction of resting blood pressure.

## Abbreviations

ANOVA: Analysis of variance
BP: Blood pressure
DBP: Diastolic blood pressure
EMG: Electromyography
HR: Heart rate
IE: Isometric exercise
IES: Isometric Exercise Scale
IET: Isometric exercise training
MAP: Mean arterial pressure
RPE: Rating of perceived exertion
SBP: Systolic blood pressure

## References

[1] Mancia G (2013). ESH/ESC guidelines for the management of arterial hypertension: The Task Force for the Management of Arterial Hypertension of the European Society of Hypertension (ESH) and of the European Society of Cardiology (ESC). Eur. Heart J..

[2] Brook RD, Jackson EA, Giorgini P, McGowan CL (2015). When and how to recommend “alternative approaches” in the management of high blood pressure. Am. J. Med..

[3] Wiles JD, Coleman DA, Swaine IL (2010). The effects of performing isometric training at two exercise intensities in healthy young males. Eur. J. Appl. Physiol..

[4] Cornelissen VA, Smart NA (2013). Exercise training for blood pressure: A systematic review and meta-analysis. J. Am. Heart Assoc..

[5] Devereux GR, Wiles JD, Howden R (2015). Immediate post-isometric exercise cardiovascular responses are associated with training-induced resting systolic blood pressure reductions. Eur. J. Appl. Physiol..

[6] Taylor KA, Wiles JD, Coleman DA, Leeson P, Sharma R, O’Driscoll JM (2018). Neurohumoral and ambulatory haemodynamic adaptations following isometric exercise training in unmedicated hypertensive patients. J. Hypertens..

[7] Robertson RJ, Goss FL, Dubé J, Rutkowski J, Dupain M, Brennan C, Andreacci J (2004). Validation of the adult OMNI scale of perceived exertion for cycle ergometer exercise. Med. Sci. Sports Exerc..

[8] Wiles JD, Taylor K, Coleman D, Sharma R, O’driscoll JM (2018). The safety of isometric exercise: Rethinking the exercise prescription paradigm for those with stage 1 hypertension. Medicine.

[9] Devereux GR, Wiles JD, Swaine IL (2010). Reductions in resting blood pressure after 4 weeks of isometric exercise training. Eur. J. Appl. Physiol..

[10] Baross AW, Wiles JD, Swaine IL (2013). Double-leg isometric exercise training in older men. Open Access J. Sports Med..

[11] McGowan CL, Levy AS, McCartney N, MacDonald MJ (2007). Isometric handgrip training does not improve flow-mediated dilation in subjects with normal blood pressure. Clin. Sci..

[12] Millar PJ, Paashuis A, McCartney N (2009). Isometric handgrip effects on hypertension. Curr. Hypertens. Rev..

[13] Goldring N, Wiles JD, Coleman D (2014). The effects of isometric wall squat exercise on heart rate and blood pressure in a normotensive population. J. Sports Sci..

[14] Wiles JD, Goldring N, O'Driscoll JM, Taylor KA, Coleman DA (2018). An alternative approach to isometric exercise training prescription for cardiovascular health. Transl. J. Am. Coll. Sports Med..

[15] Wiles JD, Goldring N, Coleman D (2017). Home-based isometric exercise training induced reductions resting blood pressure. Eur. J. Appl. Physiol..

[16] Colado JC, Garcia-Masso X, Triplett NT, Calatayud J, Flandez J, Behm D, Rogers ME (2014). Construct and concurrent validation of a new resistance intensity scale for exercise with Thera-Band^®^ elastic bands. J. Sports Sci. Med..

[17] Morrin NM, Stone MR, Swaine IL, Henderson KJ (2018). The use of the CR-10 scale to allow self-regulation of isometric exercise intensity in pre-hypertensive and hypertensive participants. Eur. J. Appl. Physiol..

[18] Chen MJ, Fan X, Moe ST (2002). Criterion-related validity of the Borg ratings of perceived exertion scale in healthy individuals: A meta-analysis. J. Sports Sci..

[19] Lagally KM, McCaw ST, Young GT, Medema HC, Thomas DQ (2004). Ratings of perceived exertion and muscle activity during the bench press exercise in recreational and novice lifters. J. Strength Condition. Res..

[20] Aniceto RR, Ritti-Dias RM, dos Prazeres TM, Farah BQ, de Lima FF, do Prado WL (2015). Rating of perceived exertion during circuit weight training: A concurrent validation study. J. Strength Condition. Res..

[21] Buckley JP, Borg GA (2011). Borg’s scales in strength training; from theory to practice in young and older adults. Appl. Physiol. Nutr. Metab..

[22] Robertson RJ, Goss FL, Andreacci JL, DubÉ JJ, Rutkowski JJ, Snee BM, Kowallis RA, Crawford K, Aaron DJ, Metz KF (2005). Validation of the children's OMNI RPE scale for stepping exercise. Med. Sci. Sports Exerc..

[23] Pincivero DM, Polen RR, Byrd BN (2010). Gender and contraction mode on perceived exertion. Int. J. Sports Med..

[24] Eston R, Evans HJL (2009). The validity of submaximal ratings of perceived exertion to predict one repetition maximum. J. Sports Sci. Med..

[25] Gearhart RF, Riechman SE, Lagally KM, Andrews RD, Robertson RJ (2011). Safety of using the adult OMNI Resistance Exercise Scale to determine 1-RM in older men and women. Percept. Mot. Skills.

[26] Borg G (1998). Perceived Exertion and Pain Scales.

[27] Robertson RJ (2003). Concurrent validation of the OMNI perceived exertion scale for resistance exercise. Med. Sci. Sports Exerc..

[28] Mays RJ, Goss FL, Schafer MA, Kim KH, Nagle-Stilley EF, Robertson RJ (2010). Validation of adult OMNI perceived exertion scales for elliptical ergometry. Percept. Mot. Skills.

[29] Whelton P.K. et al. ACC/AHA/AAPA/ABC/ACPM/AGS/APhA/ASH/ASPC/NMA/PCNA guideline for the prevention, detection, evaluation, and management of high blood pressure in adults. A Report of the American College of Cardiology/American Heart Association Task Force on Clinical Practice Guidelines. (2017).

[30] Atkinson G, Nevill AM (2001). Selected issues in the design and analysis of sport performance research. J. Sports Sci..

[31] Shrout PE, Fleiss JL (1979). Intraclass correlations: Uses in assessing rater reliability. Psychol. Bull..

[32] Field A (2009). Discovering Statistic Using SPSS for Windows.

[33] Tate RF, Klett GW (1959). Optimal confidence intervals for the variance of a normal distribution. J. Am. Stat. Assoc..

[34] Brown DM, Bray SR (2015). Isometric exercise and cognitive function: An investigation of acute dose–response effects during submaximal fatiguing contractions. J. Sports Sci..

[35] Hackett DA, Johnson NA, Halaki M, Chow CM (2012). A novel scale to assess resistance-exercise effort. J. Sports Sci..

[36] Lagally KM, Robertson RJ (2006). Construct validity of the OMNI resistance exercise scale. J. Strength Condition. Res..

[37] Desgorces FD, Thomasson R, Aboueb S, Toussaint JF, Noirez P (2015). Prediction of one-repetition maximum from submaximal ratings of perceived exertion in older adults pre-and post-training. Aging Clin. Exp. Res..

[38] Tiggemann CL, Korzenowski AL, Brentano MA, Tartaruga MP, Alberton CL, Kruel LF (2010). Perceived exertion in different strength exercise loads in sedentary, active, and trained adults. J. Strength Condition. Res..

[39] Hollander DB, Durand RJ, Trynicki JL, Larock D, Castracane VD, Hebert EP, Kraemer RR (2003). RPE, pain, and physiological adjustment to concentric and eccentric contractions. Med. Sci. Sports Exerc..

[40] Giancotti, C.f., Foster, C., Pezzotta, C., Lecce, D., Rodio, A., Capranica, L. & Cortis, C. Evaluation of training load during Suspension Training: Is session-RPE a valid method? in *Conference: 20th Annual Congress of the European College of Sport Science (ECSS)*, Malmö (Sweden), (2015).

[41] Williams MA (2007). American Heart Association Council on Clinical Cardiology; American Heart Association Council on Nutrition, Physical Activity, and Metabolism. Resistance exercise in individuals with and without cardiovascular disease: 2007 update: A scientific statement from the American Heart Association Council on Clinical Cardiology and Council on Nutrition, Physical Activity, and Metabolism. Circulation.

[42] Colado JC, Garcia-Masso X, Triplett TN, Flandez J, Borreani S, Tella V (2012). Concurrent validation of the OMNI-resistance exercise scale of perceived exertion with Thera-band resistance bands. J. Strength Condition. Res..

[43] Day ML, McGuigan MR, Brice G, Foster C (2004). Monitoring exercise intensity during resistance training using the session RPE scale. J. Strength Condition. Res..

[44] Lagally KM, Amorose AJ, Rock B (2009). Selection of resistance exercise intensity using ratings of perceived exertion from the OMNI—RES. Percept. Mot. Skills..

[45] Egan AD (2003). Session rating of perceived exertion during high intensity and low intensity bouts of resistance exercise. UW-L J. Undergrad. Res..

